# Supplementary material for: Longitudinal association between mental health and future antibiotic prescriptions in healthy adults: Results from the LOHAS
Source: PLoS One. 2020 Oct 5;15(10):e0240236. doi: 10.1371/journal.pone.0240236 (PMC7535024; doi:10.1371/journal.pone.0240236)
Supplement: S1 Table — (DOCX) [file pone.0240236.s001.docx]

**S1 Table** Adjusted odds ratios for antibiotic prescriptions, primary analysis, and sensitivity analysis

|  | Total n=816 | | MI n=967 | |
| --- | --- | --- | --- | --- |
|  | Adjusted OR (95% CI) | p value | Adjusted OR (95% CI) | p value |
| SF-12 MH score (per 1 SD) | 1.40 [1.03, 1.90] | 0.03 | 1.26 [0.96, 1.66] | 0.09 |
| SF-12 PF score (per 1 SD) | 0.95 [0.75, 1.22] | 0.71 | 0.94 [0.76, 1.16] | 0.55 |
| Age (per year) | 1.01 [0.98, 1.03] | 0.58 | 1.00 [0.98, 1.03] | 0.68 |
| Sex, female (vs. male) | 0.95 [0.52, 1.74] | 0.86 | 0.92 [0.52, 1.64] | 0.78 |
| Occupation |  |  |  |  |
| Yes (vs. no) | 1.06 [0.60, 1.86] | 0.84 | 1.06 [0.62, 1.82] | 0.82 |
| Living alone |  |  |  |  |
| Yes (vs. no) | 1.95 [0.93, 4.11] | 0.08 | 1.73 [0.84, 3.57] | 0.14 |
| Smoking status |  |  |  |  |
| Never and former smoker | Ref |  | Ref |  |
| Current smoker | 0.71 [0.31, 1.62] | 0.41 | 0.94 [0.45, 1.95] | 0.87 |
| Alcohol consumption |  |  |  |  |
| Rarely or never | Ref |  | Ref |  |
| Every day or sometimes | 1.23 [0.70, 2.15] | 0.47 | 1.26 [0.73, 2.18] | 0.41 |

MI=multiple imputation; OR=odds ratio; CI=confidence interval; SD=standard deviation; SF-12 MH=Short-Form 12 Health Survey Mental Health domain; SF-12 PF=Short-Form 12 Health Survey Physical Functioning domain; Ref=reference
