# Supplementary material for: Longitudinal association between mental health and future antibiotic prescriptions in healthy adults: Results from the LOHAS
Source: PLoS One. 2020 Oct 5;15(10):e0240236. doi: 10.1371/journal.pone.0240236 (PMC7535024; doi:10.1371/journal.pone.0240236)
Supplement: S2 Table — (DOCX) [file pone.0240236.s002.docx]

**S2 Table** Adjusted odds ratios for antibiotic prescriptions, secondary analysis, and sensitivity analysis

|  | Total n=816 | | MI n=967 | |
| --- | --- | --- | --- | --- |
|  | Adjusted OR (95% CI) | p value | Adjusted OR (95% CI) | p value |
| Depressive symptoms* with (vs. without) | 0.27 [0.11, 0.70] | 0.007 | 0.44 [0.21, 0.90] | 0.03 |
| SF-12 PF score (per 1 SD) | 0.96 [0.75, 1.22] | 0.73 | 0.94 [0.76, 1.16] | 0.55 |
| Age (per year) | 1.01 [0.99, 1.03] | 0.45 | 1.01 [0.98, 1.03] | 0.57 |
| Sex, female (vs. male) | 0.91 [0.50, 1.68] | 0.77 | 0.90 [0.51, 1.61] | 0.73 |
| Occupation |  |  |  |  |
| Yes (vs. no) | 1.09 [0.62, 1.92] | 0.76 | 1.08 [0.63, 1.85] | 0.78 |
| Living alone |  |  |  |  |
| Yes (vs. no) | 1.92 [0.91, 4.05] | 0.09 | 1.71 [0.83, 3.53] | 0.14 |
| Smoking status |  |  |  |  |
| Never and former smoker | Ref |  | Ref |  |
| Current smoker | 0.73 [0.32, 1.68] | 0.46 | 0.97 [0.47, 2.01] | 0.93 |
| Alcohol consumption |  |  |  |  |
| Rarely or never | Ref |  | Ref |  |
| Every day or sometimes | 1.21 [0.69, 2.11] | 0.51 | 1.25 [0.72, 2.17] | 0.44 |

*Depressive symptoms indicated by an SF-12 MH score ≤60

MI=multiple imputation; OR=odds ratio; CI=confidence interval; SD=standard deviation; SF-12 MH=Short-Form 12 Health Survey Mental Health domain; SF-12 PF=Short-Form 12 Health Survey Physical Functioning domain; Ref=reference
