# Supplementary material for: Longitudinal association between mental health and future antibiotic prescriptions in healthy adults: Results from the LOHAS
Source: PLoS One. 2020 Oct 5;15(10):e0240236. doi: 10.1371/journal.pone.0240236 (PMC7535024; doi:10.1371/journal.pone.0240236)
Supplement: Attachment — Submitted filename: Response to Reviewers.docx [file pone.0240236.s003.docx]

Dr. Masaki Mogi

Academic Editor

PLOS ONE

Aug 9, 2020

Re: Manuscript ID: PONE-D-20-10789

Dear Dr. Mogi,

Thank you very much for your e-mail and review of the manuscript PONE-D-20-10789 that we sent on April 15, 2020. We thank two reviewers for providing constructive comments regarding the improvement of the original manuscript. We have carefully reviewed the comments and have revised the manuscript accordingly. Our responses are given in a point-by-point manner below.

We remain very enthusiastic about publishing our original scientific article in the PLOS ONE, and look forward to your editorial decision.

Sincerely,

Yosuke Yamamoto, MD, PhD

(on behalf of all the authors)

Reviewers' comments:

Reviewer's Responses to Questions

*Comments to the Author*

1. Is the manuscript technically sound, and do the data support the conclusions?

Reviewer #1: Yes

Reviewer #2: Yes

2. Has the statistical analysis been performed appropriately and rigorously?

Reviewer #1: Yes

Reviewer #2: Yes

3. Have the authors made all data underlying the findings in their manuscript fully available?

Reviewer #1: No

Reviewer #2: Yes

Response: We have changed Data Availability Statement as follows. “The underlying data was obtained from the collaboration with the local government and contains sensitive information on individuals including gender, age, and self-reported social data, and sharing these data openly is prohibited by the contract with the local government. Data requests may be sent to iHope international (research@i-hope.jp). The authors did not have any special privileges to access the data and others would be able to access these data in the same manner as the authors.”

4. Is the manuscript presented in an intelligible fashion and written in standard English?

Reviewer #1: Yes

Reviewer #2: No

Response: The paper has been edited and rewritten by an experienced scientific editor, who has improved the grammar and stylistic expression of the paper. To clarify this, we have added acknowledgement as follows; “We would like to thank Editage (www.editage.com) for English language editing.”

**Response to Reviewer 1:**

Thank you for your review of our paper. We have answered each of your points below.

1. P5 L95: The authors call this design “retrospective” but data were collected prospectively, so I suggest to reword in order to not confuse the reader.

Response: We appreciate your suggestions. As suggested, we have changed the design as (secondary analysis of) prospective cohort study. (P2 L27, P5 L94)

2. P5 L 113: It would be useful to describe properly the selection of the study population. How many people were invited to participate? How many declined? How many excluded because of antibiotics at baseline? Or because of comorbidity? A flowchart would be very useful.

Response: We appreciate these helpful suggestions. We already submitted a flowchart as supporting files but changed it to Figure 1. (P9 L208, P10 L230) Additionally we found miscount of potential participants, we have corrected the number. (P9 L205-207)

3. P6 L122: The authors excluded participants who died or moved without any prescription. I believe this is methodologically wrong. In my opinion, the best option would be to include everyone and censor them at time of dead/emigration. Then the analysis could be performed with a Poisson model or a similar model that allows for different follow-up time in different individuals. Alternatively (if death or emigration is rare), one might exclude all those who emigrate or die (although this might bias the results). However, the authors used one approach for those with antibiotics, and another for those without. In any case, I think the same approach should be used for all study participants (this also refers to P9 L193).

Response: We thank the reviewer for the careful review of the manuscript. We agree with you. We have changed to exclude all participants with death or emigration in observation period because it was rare (only 1 died case added). The study results are almost same since additional excluded individual is only one subject. We have corrected context and all the results. (P6 L120, P9 L193)

4. P6 L133: The authors used the prescription database to identify antibiotics, but it is not clear where the disease (assumed to be treated by the antibiotics) was obtained from. Is this from another database? It would be interesting to see the concordance between the two data sources: how many are in both, how much time apart the two occurrences are, etc.

Response: We appreciate the reviewer’s suggestions and apologize for the confusion. Information on disease name and medication including antibiotics is derived from one data source. Generally, physicians are to record disease name and prescription in each visit, so we can use the integrated data. However, we could not accurately identify each antibiotics prescription to specific disease name. This is the strong limitation of this study and we have added it to the Discussion (P17 L375-377)

5. P9 L203: It would be useful to add a reference to Rubin’s rules for those unfamiliar with multiple imputation.

Response: We appreciate your suggestions. As suggested, we have added a reference below. (P9 L202, P20 L452)

Rubin DB, Schenker N: Multiple imputation in health-care databases: an overview and some applications. Stat Med 1991; 10: 585– 598.

6. P15 L323: The results of this study are clear: those with poorer mental health are less likely to be prescribed antibiotics. Have the authors considered looking only at infections? Is it possible that the rate of infections is the same but not treatment not adequate for those with mental health problems?

Response: We have performed an additional analysis with the outcome of infections for one year after answering the questionnaire. As a result, no relation was found between mental health and infections. [OR=1.11 (95%CI 0.90-1.36, p=0.34)] To explain this result, there are two possible reasons: One is that improperly excessive antibiotics were prescribed in the mentally healthy adults, which was already described in our discussion. The other is that adults with low mental health may not be properly treated infections with antibiotics, which the reviewer pointed out. However, as mentioned above, we could not accurately identify each antibiotics prescription to specific disease name in our database. This is the strong limitation of this study. To make this point clearer, we have added the Discussion (P16 L349-350, P17 L375-377)

7. P16 L338: “The absence of any significant association” should be rephrased. There is a significant association, in the other direction.

Response: We thank the reviewer for the careful review of the manuscript. We have rephrased as follows; “The unexpected association between depressive symptoms and decreased antibiotic prescriptions in this study might have been due to the excessive use of antibiotics among mentally healthy adults.” (P16 L339-341)

8. P17 L363: I think the discussion would benefit of some limitations related to the internal validity of the study. For example, is there any potential selection bias (the flow diagram mentioned above would help assess that)? Could there be some misclassification of mental health status or antibiotic use? What happens with the difference between antibiotics prescription and actually taken?

Response: We appreciate your suggestions. As suggested, we have added some limitations related to the internal validity of the study in Discussion. (P17 L373-377)

9. Finally, I have some general comments. Why did the authors decide to exclude individuals with comorbidities? Would not be possible to investigate those? Or adjust for comorbidities in the analyses? Also, what is the main idea behind excluding patients with antibiotics at baseline? I would understand this approach if causal conclusions had to be taken, for example which exposures to change to prevent future antibiotic use. However, in this study, the main aim is to describe whether individuals with poorer/better mental health are less/more likely to use antibiotics, could this question be answered with a cross-sectional design, in which establishing a temporal relation is not the main aim?

Response: We thank the reviewer for these insightful comments. In case investigating the relationship between mental health and antibiotics prescription, we consider that complications and baseline antibiotic prescribing are strong confounding factors. To exclude the effects of confounding factors, the study population was limited to adults with no complications and no baseline antibiotic prescription. Complications strongly affect mental health and future antibiotics prescription. And also, the individuals who were prescribed antibiotics at baseline are supposed to have some disease at answering questionnaire and it might cause poorer mental health and repeated antibiotic prescription.

As a subgroup analysis, we have analyzed individuals with comorbidities (Charlson comorbidity score: CCI≧1), which surprisingly showed a strong association between those with higher Physical Function (PF) and less future antibiotics prescription. (OR=0.81 95%CI=0.68, 0.95 p=0.01) We considered participants with CCI=0 and CCI≧1 were distinct properties. However, this study was originally aimed to investigate the relationship in healthy subjects without comorbidities before starting this study. Therefore, we included participants with CCI=0 only in this study.

Regarding the study design, what we wanted to know was the relationship between mental health and subsequent acute infections requiring antibiotics. That’s why we should investigate temporal relation by longitudinal study.

**Response to Reviewer 2:**

We would like to thank the reviewer for the valuable comments. Our answers to your points are as follows.

Please carefully revise the manuscript for English grammar. Otherwise I very much enjoyed reading this study, it is the first study to examine the association between mental health and antibiotic use in the community setting, a topic I have very often wondered about, thank you for your work, it should be a welcome addition to the literature.

Response: The paper has been edited and rewritten by an experienced scientific editor, who has improved the grammar and stylistic expression of the paper. To clarify this, we have added acknowledgement as follows; “We would like to thank Editage (www.editage.com) for English language editing.”
