# Supplementary material for: Longitudinal association between mental health and future antibiotic prescriptions in healthy adults: Results from the LOHAS
Source: PLoS One. 2020 Oct 5;15(10):e0240236. doi: 10.1371/journal.pone.0240236 (PMC7535024; doi:10.1371/journal.pone.0240236)
Supplement: Attachment — Submitted filename: Response to Reviewers200917.docx [file pone.0240236.s004.docx]

Dr. Masaki Mogi

Academic Editor

PLOS ONE

Sep 17, 2020

Re: Manuscript ID: PONE-D-20-10789

Dear Dr. Mogi,

Thank you very much for your e-mail and review of the manuscript PONE-D-20-10789 that we sent on April 15, 2020. We thank two reviewers for providing constructive comments regarding the improvement of the original manuscript. We have carefully reviewed the comments and have revised the manuscript accordingly. Our responses are given in a point-by-point manner below.

We remain very enthusiastic about publishing our original scientific article in the PLOS ONE, and look forward to your editorial decision.

Sincerely,

Yosuke Yamamoto, MD, PhD

(on behalf of all the authors)

Reviewer #1: Thank you for addressing all my comments.

Regarding the selection of the study population (and the included flowchart, sorry for having missed that in my first review), it would be useful to know how many were invited to participate. According to the manuscript, 984 consented to participate, but it is unknown how many declined, and this is important to assess potential selection bias. Please refer to Fig 1 also in the methods section when study population is described.

Thank you for adding text about the internal validity in the discussion. However, I think further explanations are required. I think the sentence “Selection bias might have occurred because not all residents participated” should he complemented a little bit. How many people agreed or not to participate (see my previous point)? If most people participated, then selection bias is unlikely. Do you think participation rates will be similar among those with/without antibiotic use? Or those with/without mental disorders? Selection is likely to cause bias only if associated with both exposure and outcome. Regarding information bias, do you think potential misclassification of the outcome will be differential (different among exposed and unexposed) or non-differential? If non-differential, the bias is likely to be towards the null. I think the manuscript will benefit from such thoughts in the discussion section.

**Response**: Thank you for your constructive comments. Unfortunately, we could not know the exact number of residents who refused. Invitations to our study were sent to all residents who received an annual government health check-up, but the exact number was unknown. We added such explanation in our discussion. (P17 L373) We also revised discussion with regard to measurement bias. (P17 L377) We considered the impact of measurement bias on this study was relatively small because exposure (mental health) was not associated with misclassification of antibiotic prescription.
